# ABHD11-AS1: An Emerging Long Non-Coding RNA (lncRNA) with Clinical Significance in Human Malignancies

**DOI:** 10.3390/ncrna8020021

**Published:** 2022-03-01

**Authors:** Upendarrao Golla, Kishore Sesham, Siva Dallavalasa, Naresh Kumar Manda, Sambamoorthy Unnam, Arun Kumar Sanapala, Sharada Nalla, Susmitha Kondam, Rajesh Kumar

**Affiliations:** 1 Department of Medicine, Division of Hematology and Oncology, Pennsylvania State University College of Medicine, Hershey, PA 17033, USA; 2 Penn State Cancer Institute, Pennsylvania State University College of Medicine, Hershey, PA 17033, USA; 3 Department of Anatomy, All India Institute of Medical Sciences (AIIMS), Mangalagiri 522503, India; seshamkishore@aiimsmangalagiri.edu.in; 4 Center of Excellence in Molecular Biology and Regenerative Medicine (CEMR), Department of Biochemistry, JSS Medical College, Mysuru 570015, India; sivakumar65d@gmail.com; 5 Department of Biochemistry, School of Life Sciences, University of Hyderabad, Hyderabad 500046, India; mnareshkumarpdf@uohyd.ac.in; 6 Faculty of Pharmacy, Sree Dattha Institute of Pharmacy, Ibrahimpatnam 501510, India; unnammoorthy@gmail.com (S.U.); sanapala787@gmail.com (A.K.S.); 7 Faculty of Pharmacy, University College of Pharmaceutical Sciences, Palamuru University, Mahabubnagar 509001, India; nalla.sharada@gmail.com (S.N.); susmitha.kondam@gmail.com (S.K.); 8 Department of Anatomy, All India Institute of Medical Sciences (AIIMS), New Delhi 110029, India; joyfulraj@gmail.com

**Keywords:** lncRNA, ABHD11-AS1, cancer, signaling pathways, transcription, epigenetics, PI3K/Akt, N6-methyladenosine, miRNA, biomarker

## Abstract

The aberrant expression of lncRNAs has been linked to the development and progression of different cancers. One such lncRNA is ABHD11 antisense RNA 1 (ABHD11-AS1), which has recently gained attention for its significant role in human malignancies. ABHD11-AS1 is highly expressed in gastric, lung, breast, colorectal, thyroid, pancreas, ovary, endometrium, cervix, and bladder cancers. Several reports highlighted the clinical significance of ABHD11-AS1 in prognosis, diagnosis, prediction of cancer progression stage, and treatment response. Significantly, the levels of ABHD11-AS1 in gastric juice had been exhibited as a clinical biomarker for the assessment of gastric cancer, while its serum levels have prognostic potential in thyroid cancers. The ABHD11-AS1 has been reported to exert oncogenic effects by sponging different microRNAs (miRNAs), altering signaling pathways such as PI3K/Akt, epigenetic mechanisms, and N6-methyladenosine (m^6^A) RNA modification. In contrast, the mouse homolog of AHD11-AS1 (Abhd11os) overexpression had exhibited neuroprotective effects against mutant huntingtin-induced toxicity. Considering the emerging research reports, the authors attempted in this first review on ABHD11-AS1 to summarize and highlight its oncogenic potential and clinical significance in different human cancers. Lastly, we underlined the necessity for future mechanistic studies to unravel the role of ABHD11-AS1 in tumor development, prognosis, progression, and targeted therapeutic approaches.

## 1. Introduction

**Figure 1:**
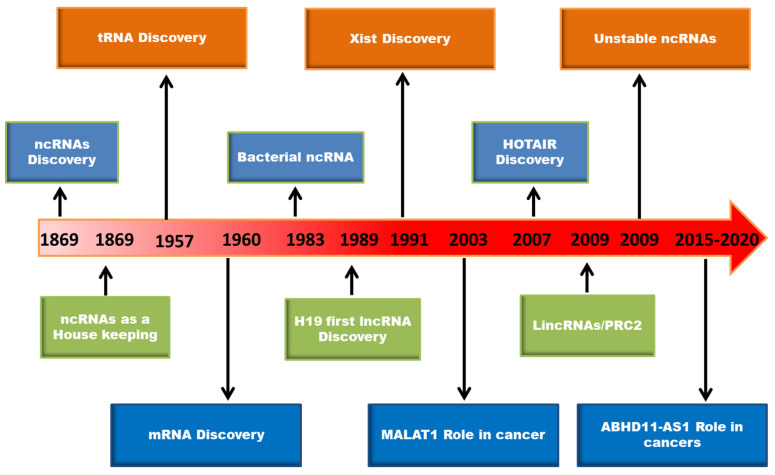
The timeline of major discoveries in the area of RNA biology.

Cancer is a dangerous disease characterized by the uncontrollable proliferation of abnormal cells with high morbidity and mortality [1]. As per WHO statistics, “cancer is the second leading cause of death globally, accounting for an estimated 9.6 million deaths, or one in six deaths, in 2018. Lung, prostate, colorectal, stomach and liver cancer are the most common types of cancer in men, while breast, colorectal, lung, cervical, and thyroid cancer are the most common among women” (https://www.who.int/ accessed on 3 January 2022). Despite enormous progress in cancer research, most cancers are diagnosed at advanced stages resulting in high mortality due to a lack of methods for early diagnosis, effective treatment, and disease relapse. The currently available cancer biomarkers for diagnosis and treatment in clinical use are proteins. Since 2% of the genome only codes for proteins, the research has been focused more on the non-coding genome. Over the past decade, elucidation of the functional importance of non-coding DNA in the human genome has gained the attention of researchers. As a result, several non-coding RNAs (ncRNAs) and long non-coding RNAs (lncRNAs) have been identified that play a critical regulatory role in various cellular processes and biological functions [2]. The timeline of significant discoveries in the field of ncRNAs is depicted in Figure 1.

**Figure 2:**
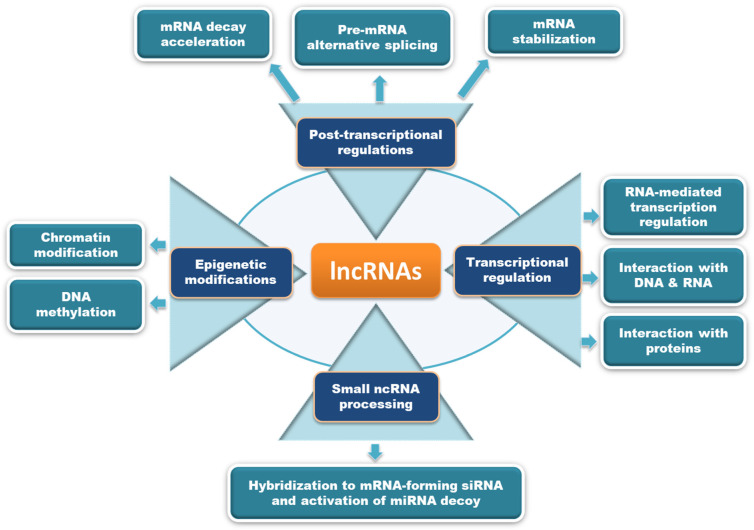
The potential mechanisms of action through which lncRNAs modulate biological functions are depicted here. LncRNAs may act either by processing small ncRNAs, epigenetic modifications, transcriptional regulation, or post-transcriptional modifications to mediate different biological effects.

LncRNAs are defined as non-protein coding RNA transcripts with over 200 nucleotides in length [3]. LncRNAs account for 80% of ncRNAs, and play important roles in various cellular functions through participating at multiple regulatory levels (transcriptional, post-transcriptional, translational, post-translational, and epigenetic) [4]. LncRNAs can interact with chromatin, RNA, and proteins to modulate the transcription of target genes. The potential mechanisms through which lncRNAs mediate their biological effects were extensively reviewed earlier, and are summarized in Figure 2 [5,6,7]. LncRNAs are categorized based on their cellular localization as nuclear lncRNAs and cytoplasmic lncRNAs. Nuclear lncRNAs are functionally enriched for chromatin interactions, transcriptional alterations, and RNA processing, whereas cytoplasmic lncRNAs modulate signaling pathways, translation, and stability of mRNA transcripts. In another way, lncRNAs can be classified based on their genomic location relative to coding genes as intronic lncRNAs, overlapping lncRNAs, bidirectional lncRNAs, sense lncRNAs, antisense lncRNAs, and long intergenic RNAs (lincRNAs), which include enhancer RNAs (eRNAs) which are transcribed from distal regulatory enhancers [8]. The aberrant regulation of lncRNAs has been associated with different diseases, including cancer [8,9]. One such lncRNA with oncogenic potential is *ABHD11* antisense RNA 1 (ABHD11-AS1; ENSG00000225969), which has gained attention over the years for its critical role in cancer progression, diagnosis, and treatment.

**Figure 3:**
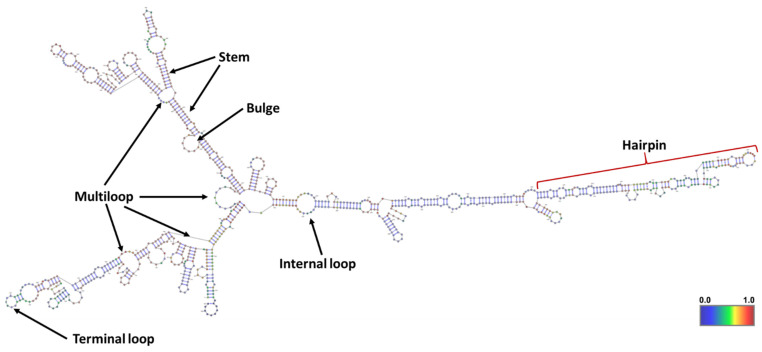
The minimum free energy (MFE) secondary structure of ABHD11-AS1 lncRNA predicted using RNAfold web server. Different components in the secondary structure of lncRNA corresponding to the topology (or shape) of base pairings were defined.

ABHD11-AS1 is an RNA gene known as long intergenic non-protein coding RNA 35 (LINC00035) and Williams–Beuren syndrome chromosome region 26 (WBSCR26), located at 7q11.23. Firstly, ABHD11-AS1 was reported to upregulate and correlate with clinicopathological features in gastric cancer progression [10]. After that, ABHD11-AS1 gained researchers’ attention to exploit its role in the development, progression, and prognosis of breast, colorectal, lung, thyroid, pancreatic, bladder, endometrial, cervical, and ovarian cancers. In some studies, ABHD11-AS1 was found as circulatory lncRNA that serves as a molecular marker for the early diagnosis of cancers. Several mutations in ABHD11-AS1 lncRNA were reported, and their impact on its overall structure and function needs to be investigated. Here, the secondary structure of ABHD11-AS1 lncRNA predicted using RNAfold web server is depicted in Figure 3 [11]. Considering the increased attention on biological significance of lncRNAs, we attempted in this first review on ABHD11-AS1 to summarize the oncogenic effects and mechanisms through which it contributes to tumor development, progression, invasion, metastasis, diagnosis, and treatment.

## 2. Oncogenic Role of ABHD11-AS1 lncRNA in Human Cancers

Among numerous lncRNAs, a handful were characterized as they were very poorly expressed, poorly conserved, and more tissue specific. Existing literature indicates that some lncRNAs function in ‘trans’, far from their site of transcription, while others act in ‘cis’, modulating the expression of nearby genes. Some lncRNAs play functional roles in cellular homeostasis, growth, differentiation, and act as oncogenes or tumor suppressors [12]. Though the functions of ABHD11-AS1 lncRNA in healthy tissues are not characterized, a recent comprehensive analysis of genotype–tissue expression (GTEx) datasets revealed its expression in spleen and blood tissues [13]. The mouse homolog of ABHD11-AS1 (Abhd11os) was expressed in striatal neurons, and significantly downregulated in Huntington disease (HD) models. Overexpression of Abhd11os showed neuroprotection against an N-terminal fragment of mutant huntingtin, while knockdown was protoxic [14]. Recent pan-cancer comparison revealed that lncRNAs were deregulated more than tumor-specific than mRNAs [13]. Most of the deregulated lncRNAs were tumor-specific, while few of them act as “onco-lncRNAs”, which are dysregulated across several cancer types [15]. ABHD11-AS1 is one of such onco-lncRNAs that was overexpressed in different cancers such as gastric cancer (GC), papillary thyroid cancer (PTC), non-small cell lung cancer (NSCLC), pancreatic cancer (PC), colorectal cancer (CRC), ovarian cancer (OC), breast cancer (BC), endometrial cancer (EC), cervical cancer (CC), and bladder cancer, as summarized in Table 1 and discussed in detail as follows.

### 2.1. Gastric Cancer

Gastric cancer (GC) is the most common gastrointestinal malignant tumor, with a high mortality and recurrence rate, typically originating from the gastric mucosal epithelium [38]. GC is the third leading cause of death after lung cancer and liver cancer-related mortalities globally. Over 1 million new GC cases are diagnosed annually, and nearly 0.8 million deaths, accounting for ~10% of all cancer-related deaths worldwide [1]. GC development is closely associated with several factors, including genetic mutation, helicobacter pylori infection, smoking, or intake of salty food [39,40]. With the rise in GC incidence and mortality, specifically in low-and middle-income countries, there is a need to explore underlying mechanisms of GC development, early diagnosis methods, and effective therapeutic targets. Accumulating evidence highlights the role of lncRNAs in the development and progression of GC [41,42,43]. ABHD11-AS1 is one of such lncRNAs studied explicitly and projected as a potential biomarker for early diagnosis and treatment of GC [10,16,18]. In 2014, the expression analysis showed that ABHD11-AS1 was upregulated in GC tissues from 75 patients compared to normal tissues. The ABHD11-AS1 expression levels in GC tissues were significantly related to the degree of differentiation, Lauren histologic classification, and carbohydrate antigen 19-9 (CA199) of GC patients [10]. Later, the same research group revealed that the high expression level of ABHD11-AS1 in gastric juice from GC patients could serve as a potential biomarker for the early diagnosis of GC [16]. The authors have quantified ABHD11-AS1 expression in 173 tissue samples and 130 gastric juices from different stages of gastric tumorigenesis. The levels of ABHD11-AS1 in 73 GC tissues were significantly higher than 37 healthy gastric mucosa, 34 benign lesions, and 29 gastric dysplasia. Moreover, the expression levels of ABHD11-AS1 in gastric juice from 39 GC patients were significantly greater than that from 45 normal mucosa or minimal gastritis (NMMG), 30 gastric ulcers, and 16 atrophic gastritis cases. ABHD11-AS1 levels in gastric juice from GC patients were associated with gender, tumor size and stage, Lauren classification, and blood carcinoembryonic antigen (CEA). The early GC detection rate is approximately 72% when gastric juice ABHD11-AS1 levels are used as a marker [16].

The expression levels of ABHD11-AS1 lncRNA in the plasma of GC patients of different stages were not high enough to differentiate from that of healthy control subjects [17]. Very recently, Xin et al. have exploited the function and mechanism of ABHD11-AS1 in the development and progression of GC [18]. Similar to previous reports, ABHD11-AS1 was upregulated in GC tissues and GC cell lines (SGC-7901, MKN28, AGS, MGC-803, and BGC-823), relative to the matched normal tissues and normal gastric cell lines (GES-1 and RGM-1), respectively. The siRNA-mediated knockdown of ABHD11-AS1 in gastric cell lines (MGC-803 and BGC-823) hampered the proliferation, and enhanced the apoptosis in vitro, and inhibited tumor progression in GC xenograft models in vivo [18]. Thus, ABHD11-AS1 was established as a potential therapeutic target and biomarker for the early diagnosis and treatment of GC.

### 2.2. Papillary Thyroid Cancer

Thyroid cancer is the most typical endocrine-related malignant tumor. The incidence and mortality rate of thyroid cancer patients has continuously increased for several decades, with approximately 300,000 new cases and 40,000 deaths every year worldwide [44]. Thyroid cancer is histologically subtyped into differentiated (papillary and follicular thyroid carcinoma) and anaplastic thyroid carcinoma. Papillary thyroid carcinoma (PTC) is the primary subtype, accounting for 85–90% of thyroid cancer cases [45]. Several investigations are underway to study molecular mechanisms behind the pathogenesis of thyroid cancer, and to identify potential biomarkers for early diagnosis and effective treatment of PTC. Recently, the lncRNAs were found to play critical roles in the initiation and progression of cancers, including thyroid cancer [46,47]. Several studies have reported that ABHD11-AS1 lncRNA is vital for PTC progression, and could be a potential therapeutic target [19,20,21,22]. In these studies, ABHD11-AS1 is highly expressed in PTC patient samples and cell lines (BCPAP, BHP2-7, BHP5-16, BHT-101, GLAG-66, IHH-4, K-1, KTC-1, and TPC-1), compared to normal tissues and the control cell line (Nthy-ori 3-1) respectively. The high levels of ABHD11-AS1 significantly correlated with PTC clinicopathological features, such as extrathyroidal extension, lymph node metastasis, tumor infiltration, tumor size, and advanced TNM (Tumor, Node, Metastasis) stage [19,20,22]. Kaplan–Meier analysis revealed the predictive value of ABHD11-AS1 in PTC, as the patients with higher ABHD11-AS1 had poorer overall survival [19]. Moreover, Hou et al. reported that serum ABHD11-AS1 levels were elevated in PTC patients than healthy controls, and closely correlated only with tumor diameter and lymph node metastasis [21]. The higher expression of serum ABHD11-AS1 remarkably related to poor prognosis group (patients suffered from recurrence or progression of PTC or died) that exhibited a lower five-year overall survival rate. The Cox analysis revealed TNM staging, lymph node metastasis, and ABHD11-AS1 were independent prognostic factors for PTC [21]. Furthermore, the reduction of ABHD11-AS1 levels exhibited slower proliferation, invasion, metastasis with higher apoptosis in PTC cell lines in vitro, and decreased in vivo tumor progression in PTC xenograft models [19,20]. The PTC cell lines BCPAP [19] and TPC-1 [20], transfected with ABHD11-AS1 shRNA and then injected subcutaneously into nude mice, had exhibited a drastic reduction in tumor growth rate, weights, and sizes, compared to that of empty vector control xenografts. Interestingly, xenografts with ABHD11-AS1 silencing had suppressed the liver and lung metastasis in vivo [19]. Therefore, ABHD11-AS1 exerts malignant properties in PTC progression, and its serum levels could serve as a potential biomarker for the diagnosis and treatment of PTC.

### 2.3. Ovarian Cancer

Ovarian cancer (OC) represents the most lethal gynecological cancer of the female reproductive organs. Epithelial ovarian cancer (EOC) is prevalent, and accounts for more than 90% of OC cases worldwide [48]. EOC is the leading cause of OC deaths due to extensive peritoneal metastasis, high relapse rate, a lack of specific symptoms, and a lack of reliable methods for early diagnosis. In spite of current developments in chemotherapy and cytoreductive surgery, the five-year overall survival is approximately 30%, with the prognosis of OC remaining very poor [49]. In search of potential biomarkers, lncRNAs have emerged as critical players in the development and progression of various cancers, including OC/EOC [50,51]. ABHD11-AS1 lncRNA was proven to be a potential candidate biomarker for OC/EOC by several studies [30,31,32]. The lncRNA ABHD11-AS1 was significantly overexpressed in expression analysis of EOC [30,31] and OC [32] patient samples, compared to the normal tissue controls. A similar trend of ABHD11-AS1 expression was observed in EOC (HO8910, OVCA429) and OC cell lines (A2780, HEY, SKOV-3, and OVCAR-3), relative to the normal ovarian epithelial cell line (IOSE80) and normal ovarian cells (HOSEpiC). The expression of ABHD11-AS1 was positively correlated with the lymph node metastasis and tumor stage, while negatively affecting the overall survival rate in OC patients. The knockdown of ABHD11-AS1 in EOC/OC cell lines exhibited decreased proliferation, invasiveness, and migration with more apoptosis. In comparison, the overexpression of ABHD11-AS1 resulted in opposite effects in vitro [30,31,32]. The oncogenic potential of ABHD11-AS1 lncRNA was evaluated in different xenograft models of OC in vivo [30,31]. The OC xenograft was constructed in nude mice by intraperitoneal injection of the OC cell line. Intraperitoneal delivery of ABHD11-AS1 shRNA (shABHD11-AS1) into OC xenografts suppressed in vivo tumor growth, as evidenced by reduced tumor weights and volumes, compared to mock controls [31]. In another study, OC xenografts were generated in BALB/c nude mice by injecting 10 million A2780 cells transfected with lncRNA ABHD11-AS1 (or mock control) subcutaneously or intraperitoneally [30]. Remarkably, tumor xenografts showed increased tumor growth rate and tumor volumes upon lncRNA ABHD11-AS1 overexpression, compared to mock controls. The xenografts exhibited significantly widespread metastatic lesions among mesentery in the ABHD11-AS1 overexpression group, relative to the control [30]. Thus, the current evidence strongly indicates that ABHD11-AS1 could be a potential prognostic factor for OC.

### 2.4. Colorectal Cancer

Colorectal cancer (CRC) is the most frequently diagnosed digestive system malignant tumor, and the leading cause of cancer deaths worldwide [52]. CRC is developed by the contribution of several genetic, epigenetic, and environmental factors. Although there has been tremendous progress in screening and treatment, the prognosis and five-year overall survival rate are still poor due to recurrence, distant metastasis, and limitations in the methods for early diagnosis [53]. Over the past decade, lncRNAs have gained a lot of attention due to their potential as a reliable and valid biomarker for early diagnosis, treatment, and drug resistance in different cancers, including CRC [54,55]. Several studies have reported the significance of ABHD11-AS1 lncRNA in the development, progression, and invasion of CRC [27,28,29]. Consistently, ABHD11-AS1 was highly expressed in CRC patient samples and various cell lines (SW-480, HT-29, LoVo, HCT-116, HCT-8, SW-620, Caco-2) relative to the normal controls. Kaplan–Meier survival analysis with log-rank tests revealed that CRC patients with higher ABHD11-AS1 lncRNA expression had significantly poorer progression-free survival and overall survival than those with lower levels. Moreover, the expression levels of ABHD11-AS1 are clinically associated with TNM stage and lymph node metastasis in CRC patients. The CRC cell lines (SW-480, HCT-116) exhibited a considerable reduction in the proliferation, migration, and invasion with an increase in the apoptosis upon si-RNA [27,28] or shRNA [29] targeted knockdown of ABHD11-AS1 in vitro. Accordingly, the knockdown of ABHD11-AS1 impaired the in vivo growth of SW-480 [28] and HCT-116 [29] cell lines in the xenograft model. The CRC xenografts had shown a significant reduction in tumor growth and size upon ABHD11-AS1 knockdown, relative to the control [28,29]. The findings from multiple reports highlighted the oncogenic role of ABHD11-AS1 lncRNA in CRC progression and prognosis.

### 2.5. Pancreatic Cancer

Pancreatic cancer (PC) is the seventh most common malignant tumor worldwide, with high mortality due to lack of specific symptoms, high invasiveness, and metastasis. PC can develop in either exocrine cells or neuroendocrine cells (islet cells) of the pancreas. The exocrine cancers are most common in PC, and diagnosed at an advanced stage, whereas pancreatic neuroendocrine tumors (NETs) or islet cell tumors are less familiar with a better prognosis. Despite the current research and therapeutic development progress, the median survival time is only 3–6 months, with a five-year survival rate remaining under 5% [56,57]. The lack of effective methods for early PC detection is a prime reason for the poor survival rate. As of today, lncRNAs have been exploited as the biomarkers for the clinical diagnosis and treatment of various cancers, including PC [58,59]. One such circulatory lncRNA is ABHD11-AS1, which was recently studied for its significance in PC progression, diagnosis, and treatment [24,25,26]. In 2018, Qiao X. et al. reported that ABHD11-AS1 was significantly upregulated in different PC cell lines (CFPAC-1, BXPC-3, L3.6pl, and PANC-1) and 147 PC patient tissues in comparison to pancreatic cell line (HPDE6-C7) and matched normal pancreatic tissues, respectively [24]. Interestingly, the high expression of ABHD11-AS1 correlated clinically with distant metastasis, TNM stage, tumor differentiation, and a five-year reduction in overall survival of PC patients. Similarly, the analysis of TCGA gene expression data for PC also revealed that ABHD11-AS1 was highly expressed and negatively correlated with survival rate in patients with PC [26].

Moreover, the knockdown of ABHD11-AS1 in three PC cell lines (L3.6pl, MiaPaCa-1, and PANC-1) resulted in decreased cell proliferation, colony formation, and metastasis (migration and invasion) with increased apoptosis [24,26]. Targeted knockdown of ABHD11-AS1 significantly repressed the in vivo growth of PANC-1 cells injected subcutaneously into a nude mice xenograft model [26]. Liu Y. et al. identified that the expression levels of circulatory ABHD11-AS1 lncRNA alone or combined with carbohydrate antigen 19-9 (CA199) in the plasma are potential biomarkers for early detection of PC [25]. Thus, ABHD11-AS1 plays a critical role in the diagnosis, progression, metastasis, and treatment of PC. Further studies to test the part of ABHD11-AS1 in large-scale PC patient cohorts are necessary to validate its clinical significance.

### 2.6. Luminal Breast Cancer

Breast cancer (BC) is a common cancer among women, accounting for 30% of all female cancer cases reported worldwide. BC is the second leading cause of cancer-related deaths [60]. The luminal subtype of BC is characterized by estrogen and progesterone receptors’ expression, and accounts for more than 70% of BC. The luminal subtype of BC is further classified as luminal A or luminal B, based on the levels of human epidermal growth factor receptor 2 (HER2) and ki-67 [61]. The emerging reports indicate that lncRNAs are dysregulated in BC, and the differential expression of different lncRNAs in BC subtypes has been explored [62]. One such lncRNA is ABHD11-AS1, which has been studied for its expression levels and clinical correlation in BC, specifically luminal subtype, using bioinformatics and systems biology analyses [34]. The authors have tested the expression of ABHD11-AS1 lncRNA by qPCR, and discovered that it is significantly upregulated in 79 luminal BC tissues and cell lines (T47D and MCF7), compared to the normal controls. However, the authors failed to find a considerable correlation between ABHD11-AS1 expression levels and luminal BC clinicopathological features. Accordingly, no significant association was observed with ABHD11-AS1 lncRNA expression with the overall survival of the luminal BC patients in Kaplan–Meier analysis. Thus, ABHD11-AS1 levels were not suggested as a promising biomarker for diagnosing luminal subtypes of BC. The International Cancer Genome Consortium (ICGC) portal results indicated 17 mutations in ABHD11-AS1 across luminal BC, of which one is insertion, and 16 are substitutions, with only one donor out of 19,729 (0.01%) affected. Out of 17 reported mutations, 10 of them occurred upstream, and 6 of them were located downstream of ABHD11-AS1, with only one substitution in the exon region (chr7:g.73149393A>T) [34]. Since the substitution mutations affect the secondary structure of lncRNAs and contribute to the development of cancers, further detailed studies are necessary for understanding the functional consequences of ABHD11-AS1 mutations [63]. Bioinformatic analysis of genes co-expressed with ABHD11-AS1 lncRNA are involved in signal transducer activity, transmembrane transport, reproductive process, Wnt ligand biogenesis, mesodermal commitment pathway, FGFR1 mutant receptor activation, and Wnt signaling. The protein–protein interactions (PPI) network analysis of the ABHD11-AS1 co-expressed genes identified four (*LACTB2*, *SPANXA1*, *SPANXA2*, and *SPANXC*) hub genes [34]. Recently, Wang X. et al. integrated gene co-expression network analysis in BC with clinical data, and revealed ABHD11-AS1 as a potential biomarker or BC–related risk target in several prognostic modules [64]. ABHD11-AS1 lncRNA was identified as a critical gene with significantly higher expression status in the BC survival–related modules. The prognostic module consists of ABHD11-AS1 in combination with other clinical indicators, such as estrogen receptor (ER), progesterone receptor (PR), and human epidermal growth factor receptor 2 (HER2), and TNM, had shown high accuracy and sensitivity for BC risk-stratification [64]. Although the bioinformatics analysis identified the possible role of lncRNA, further studies are warranted to prove the clinical significance of ABHD11-AS1 in the prognosis and treatment of luminal BC.

### 2.7. Non-Small Cell Lung Cancer

Lung cancer incidence and mortality have increased over the past decade globally. Non-small cell lung cancer (NSCLC) is the most common histological type, accounting for 85% of reported lung cancer cases and deaths. Despite the tremendous progress in NSCLC diagnosis and treatment regimen over recent years, the five-year survival rate of NSCLC patients remains around 15% [65]. Therefore, the development of early diagnosis methods and new therapeutic targets would be the most effective approach for reducing NSCLC deaths. The emerging reports have been highlighted that lncRNAs play a critical role in the occurrence, progression, metastasis, and chemotherapy resistance of several cancers, including NSCLC [4]. Incredibly, several lncRNAs with important biological functions aberrantly expressed and detected in the peripheral body fluids would be ideal biomarkers for the early detection of NSCLC [66]. One such circulating ABHD11-AS1 lncRNA with prognostic value in different cancers was recently studied in NSCLC by Xue L. et al. [23]. The authors have found that ABHD11-AS1 is upregulated in NSCLC cell lines (NCI-H1299, A549, HCC827, and NCI-H1650), compared to normal lung epithelial cells (BEAS-2B). The qPCR expression analysis using 40 NSCLC tissue specimens showed significant overexpression of ABHD11-AS1 compared with the adjacent normal lung tissue. Notably, ABHD11-AS1 expression was much higher in the advanced tumor stage (TNM grading) than in the primary tumor stage NSCLC tissue specimens. Accordingly, the overall survival rate was significantly lower in the NSCLC patient group with higher ABHD11-AS1 levels. Furthermore, the shRNA targeted silencing of ABHD11-AS1 decreased the proliferation of the H1299 cell line by reducing glucose uptake, lactate production, and ATP accumulation. The extracellular acidification rate (ECAR) analysis showed that ABHD11-AS1 knockdown reduced the glycolytic capacity of cells. The overexpression of ABHD11-AS1 in the H1650 cell line exhibited contrary effects to knockdown, as expected. Altogether, the authors established the oncogenic role of ABHD11-AS1 lncRNA that promotes the Warburg effect in NSCLC [23]. Further mechanistic studies are warranted to detect ABHD11-AS11 in the peripheral circulation of NSCLC patients to highlight its diagnostic value.

### 2.8. Bladder Cancer

Bladder cancer is the ninth most commonly diagnosed cancer globally, with an estimate of 500,000 cases annually. Since 2017, several studies have reported aberrant expression of lncRNAs that play a functional role in the prognosis, migration, and invasion of bladder cancer [67,68,69]. Recently, Su G. et al. performed a systematic meta-analysis of lncRNAs aberrantly regulated in bladder cancer, and discovered that UCA1 lncRNA could serve as a potential marker for bladder cancer diagnosis [70]. Chen M. et al. studied the functional impact of emerging ABHD11-AS1 lncRNA in bladder cancer tissues and cell lines in vitro [33]. The authors first established that ABHD11-AS1 was upregulated in 66 bladder cancer patient tissues and cell lines relative to normal healthy controls. Interestingly, the upregulation of ABHD11-AS1 had a significant association with clinical pathologic grading (*p* < 0.001), tumor invasion depth (*p* = 0.001), and TNM stage. The authors have implicated different bladder cancer cell lines (T24, 5637, and SW780), which had higher ABHD11-AS1 expression than SV-HUC-1, a normal control cell line established from healthy ureter tissue. The siRNA-mediated knockdown of ABHD11-AS1 resulted in decreased cell proliferation (in MTT and EdU assays) and cell migration (scratch assay), along with enhanced apoptosis (Caspase-3 activity and flow cytometry) in bladder cancer cell lines. The overexpression of ABHD11-AS1 in bladder cancer cell lines exhibited contrary effects to its silencing. Considering these in vitro results, authors have concluded that ABHD11-AS1 lncRNA serves as an oncogene, and might be a potential therapeutic target in bladder cancer [33]. Further in vivo studies are necessary with more patient samples and mechanistic insights to explore the prognostic value of ABHD11-AS1 lncRNA in bladder cancer.

### 2.9. Endometrial Cancer

Endometrial cancer (EC) is also called uterine cancer, in which malignant cells form in the endometrium tissue lining of the uterus. The incidence of EC is increasing every year, and leads to the second highest rates of death from gynecological cancers in women worldwide. EC is diagnosed at an average age of 60, and primarily affects post-menopausal women [71]. In recent years, the contribution of lncRNAs to cancer progression and their potential as biomarkers and therapeutic targets in EC has been explored [72,73]. One such lncRNA is ABHD11-AS1, which was reported to promote EC development and progression by Liu Y. et al. in 2018 [35]. In this study, the authors have tested ABHD11-AS1 expression in 89 EC tissues collected from patients undergoing surgical resection and 27 normal endometrial specimens. Interestingly, the real-time PCR analysis revealed that ABHD11-AS1 expression was significantly higher in EC tissues than in normal endometrial tissues. The knockdown of ABHD11-AS1 using siRNA in EC cell line HEC-1B (with high ABHD11-AS1 levels) resulted in decreased cell proliferation, migration, invasion, and G1 phase arrest in the cell cycle, with increased apoptosis. Overexpression of ABHD11-AS1 in the EC cell line Ishikawa (with low ABHD11-AS1 levels) resulted in increased cell proliferation, invasion, and migration, with G1-S progression in cell cycle and decreased apoptosis in vitro. Moreover, the authors have established the role of ABHD11-AS1 in EC progression in vivo using a xenograft model. The EC xenograft mice injected with Ishikawa cells overexpressing ABHD11-AS1 showed higher tumor volumes compared to mock injected control mice. The authors have established that ABHD11-AS1 promotes cell proliferation and invasion in EC [35]. Further studies are encouraged to elucidate the molecular mechanisms, through which ABHD11-AS1 contributes to EC development and progression.

### 2.10. Cervical Cancer

Cervical cancer (CC) is the primary gynecological malignant tumor that develops in a woman’s cervix. As per WHO statistics of 2018, 570,000 were diagnosed, and 300,000 women have died from CC globally. Mostly, the infection with high-risk human papillomaviruses (HR-HPV) has been linked to CC cases. In addition to HR-HPV infection, an individual’s genetic and epigenetic alterations significantly contribute to the development of CC [74,75]. The recent literature demonstrated the critical role of lncRNAs as biomarkers for CC development, invasion, metastasis, treatment response, and drug resistance [76,77]. Hou S. et al. have established the role of ABHD11-AS1 lncRNA in the progression of CC [36]. The authors found that ABHD11-AS1 was highly expressed in four different CC cell lines (HCC94, HeLa, C-33A, and CaSki) compared to the normal endocervical End1/E6E7 cell line initially. Later, the knockdown of ABHD11-AS1 using its specific shRNAs in CC cell lines C-33A and CaSki exhibited decreased cell proliferation by EdU assay and increased apoptosis by TUNEL assay. The results from the transwell assay depicted that the migration and invasion of CC cell lines decreased upon ABHD11-AS1 knockdown. Accordingly, the depletion of ABHD11-AS1 in the CaSki CC cell line significantly suppressed tumor growth and metastasis in vivo using the CC xenograft model [36]. Lately, Zhu D. et al. had established that ABHD11-AS1 levels were higher in the serum and tissues of CC patients than healthy controls, and significantly associated with the poorer prognosis and three-year overall survival of CC patients [37]. ABHD11-AS1 knockdown in HeLa and CaSki CC cell lines resulted in slower growth, reduced invasion, and metastasis with higher apoptosis, while overexpression of ABHD11-AS1 showed contrary effects in vitro [37]. Altogether, these studies had established that ABHD11-AS1 lncRNA levels could serve as a biomarker for CC diagnosis and correlate with prognosis, metastasis, and treatment in CC patients.

## 3. Molecular Mechanisms of ABHD11-AS1 lncRNA Dysregulation in Human Malignancies

**Figure 4:**
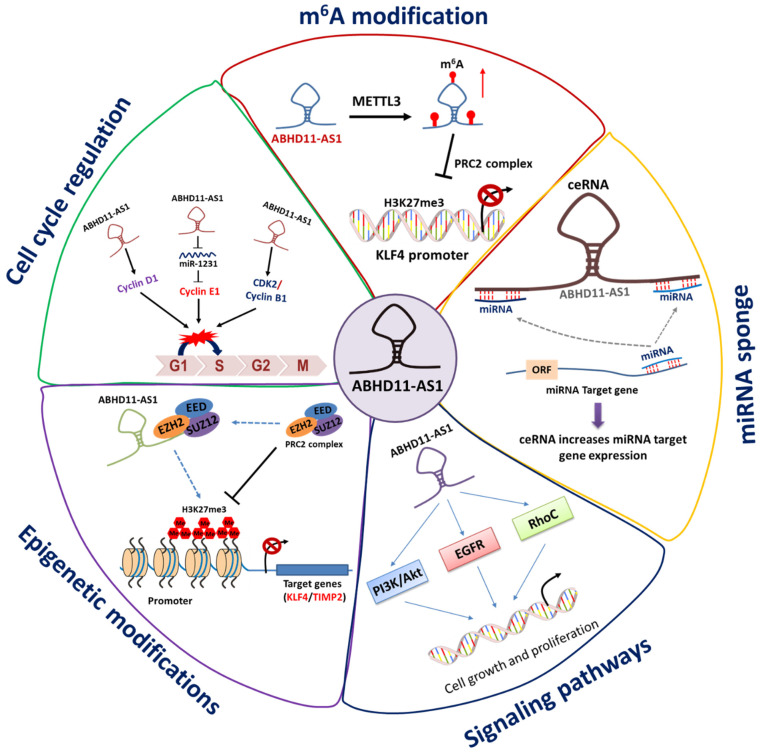
The molecular mechanisms of ABHD11-AS1 lncRNA play critical role in contributing to tumorigenesis and progression. ABHD11-AS1 act as a ceRNA (sponge miRNAs), undergoes m^6^A modification, and modulates the cell cycle, signaling pathways, and epigenetic modifications to regulate biological functions.

This section discusses various regulatory mechanisms through which ABHD11-AS1 contributes to the development and progression of different types of cancers. The modes of action of ABHD11-AS1 lncRNA are summarized in Figure 4, and discussed as follows.

### 3.1. ABHD11-AS1 Sponges Multiple miRNAs to Promote Cancer Progression

miRNAs are a class of small non-coding RNAs (sncRNAs) of 19–24 nucleotides in length, which can post-transcriptionally repress gene expression via binding to the 3’-untranslated region (3’-UTR) and translational inhibition of multiple target mRNAs and/or other RNAs [78]. LncRNAs are denoted as competitive endogenous RNAs (ceRNAs) if they act as miRNA sponges that mimic endogenous mRNA targets and compete with miRNAs for regulating gene expression [79]. ABHD11-AS1 lncRNA is one of such ceRNAs that act as a sponge for multiple miRNAs listed in Table 2 to modulate target gene expression, thus contributing to the tumor development and progression.

Lei et al. reported that ABHD11-AS1 interacts with miR-133a, which acts as a tumor suppressor in CRC by targeting LIM, SH3 protein 1, and inhibiting the mitogen-activated protein kinase (MAPK) pathway [27,80]. Among different miRNAs with putative binding sites for ABHD1-AS1, miR-133a/b showed a significant upregulation relative to control in pull-down assay using a biotinylated ABHD11-AS1 DNA probe. miR-133a had exhibited a negative correlation with ABHD11-AS1 expression levels in CRC patients and cell lines. In a dual-luciferase reporter assay, miR-133a mimic significantly attenuated the luciferase activity of the wild-type ABHD11-AS1, while it did not in that of mutated version. The authors further confirmed by RNA immunoprecipitation (RIP) assay using Ago2 antibody that ABHD11-AS1 sponges miR-133a with the implication of RNA-induced silencing complex (RISC). The developmental transcriptional factor *SOX4* (SRY-Box Transcription Factor 4) was a potential downstream target of miR-133a and ABHD11-AS1/miR-133a/*SOX4* axis established as crucial for the prognosis of CRC [27]. Accordingly, ABHD11-AS1 sponges miR-1254 to regulate the expression and signaling mediated by its downstream target, WNT11, thus promoting the progression of CRC [28]. Moreover, miR1254 significantly downregulated and related to other clinicopathologic features such as age, HPV, tumor size, histology, and histology grade of CC [81]. Lately, Zhu D. et al. found that miR-1254 levels in serum and tissues of CC were inversely correlated to that of ABHD11-AS1 expression, and significantly associated with the overall three-year follow-up prognosis of CC patients [37].

ABHD11-AS1 was found to sponge miRNAs miR-1301-3p and miR-199a-5p in PTC, and modulated the expression of their downstream targets *STAT3* and *SLC1A5*, respectively [19,20]. In a dual-luciferase reporter assay, miR-199a-5p plasmid transfection significantly attenuated the luciferase activity of the wild-type, while it did not in that of a mutated version of ABHD11-AS1 in PTC cells. Spearman’s correlation analysis showed a negative relationship between ABHD11-AS1 and miR-199a-5p expression levels in PTC tissues and cells [20]. The miRNA miR-133a-3p was used as a sponge by ABHD11-AS1 to promote the progression of OC [32]. Lysophosphatidic acid receptor 3 (*LPAR3*) and epidermal growth factor receptor (*EGFR*) were direct targets of miR-133a-3p in thyroid cancer [82]. However, the direct target of ABHD11-AS1/miR-133a-3p in OC still needs to be investigated. ABHD11-AS1 sponges miR-361-3p [18], miR-1231 [26], and miR-330-5p [36] to exhibit oncogenic effects via targeting *PDPK1*, *CCNE1* (cyclin E1), and *MARK2* in GC, PC, and CC, respectively. The current literature indicates that ABHD11-AS1 acts as ceRNA by sponging miRNAs to exert oncogenic effects in human cancers, and could be a potential therapeutic target.

### 3.2. Targeting of Key Oncogenic Signaling Pathways by ABHD11-AS1

LncRNAs are known to regulate several oncogenic signaling pathways that control proliferation, growth, metastasis, invasion, and apoptosis by modulating critical signaling components and pathway activation [8,83]. The signaling pathways targeted by ABHD11-AS1 lncRNA to exert oncogenic effects are discussed in the following sections. 

#### 3.2.1. Phosphoinositide 3 Kinase (PI3K)/Akt Signaling Pathway

PI3K/Akt signaling is a vital pathway that regulates various cellular processes, including proliferation, differentiation, survival, and migration. This pathway is most frequently altered signaling in human cancers development and progression [84]. It has been validated that lncRNAs mediate oncogenic effects very often through activating this PI3K/Akt signaling pathway, and the phosphorylation of Akt regulates tumor growth and metastasis through activating its downstream effectors [85]. Likewise, ABHD11-AS1 was reported to promote the progression of different cancers through activating PI3K/Akt signaling pathway. The knockdown of ABHD11-AS1 significantly downregulated PI3K/Akt signaling pathway in PC [24] and PTC via miR-1301-3p/*STAT3* axis [19], thus repressing the tumor progression, metastasis, and invasion. Similarly, ABHD11-AS1 was shown to activate PI3K/Akt signaling pathway through miR-361-3p/*PDPK1* axis, and contribute to GC progression and metastasis [18]. Very recently, both bioinformatic analysis and Western blotting analysis revealed that PI3K/Akt pathway is the potential signaling pathway targeted by ABHD11-AS1 to promote proliferation, migration, and invasion in CRC [29]. Thus, it is well established that ABHD11-AS1 lncRNA primarily activates PI3K/Akt signaling pathway to exert oncogenic effects in several cancers.

#### 3.2.2. Epidermal Growth Factor Receptor (EGFR) Signaling Pathway

The trans-membrane EGFR proteins are a family of receptor tyrosine kinases activated upon binding with peptide growth factors of the EGF-family of proteins. Due to genomic alterations, high expression and constitutive activation of EGFR often correlate positively with uncontrolled cell proliferation, tumor progression, and metastasis in human cancers, including OC, leading to a poor prognosis [86,87]. The anticancer therapeutics targeting EGFR signaling pathway have been developed, including erlotinib, gefitinib, and icotinib [88]. High expression of ABHD11-AS1 lncRNA correlated positively with EGFR levels in both EOC tissues and cell lines [31]. The knockdown of either ABHD11-AS1 or EGFR in EOC cell lines (HO8910, OVCA429) resulted in decreased cell growth, migration, and invasion with altered epithelial–mesenchymal transition (EMT) markers. The authors also found the upregulation of ABHD11-AS1 through the activated EGFR pathway via STAT3 for progression of EOC [31]. Analysis of TCGA datasets revealed that EGFR signaling pathway genes were highly expressed and enriched in PTC. Lu H. et al. showed that knockdown of ABHD11-AS1 resulted in downregulation of Epidermal Growth Factor Receptor Pathway Substrate 15 Like 1 (*EPS15L1*) [22]. A positive correlation between ABHD11-AS1 levels and the EGFR signaling pathway was observed in PTC cell lines. Further studies are necessary to define the collaboration between EGFR signaling and ABHD11-AS1 in other human malignancies.

#### 3.2.3. Ras Homolog Gene Family Member C (RhoC) Signaling Pathway

RhoC is one of the Rho family of small Guanosine Triphosphatases (GTPases), which are key players regulating the cytoskeletal organization and affect cell division, migration, and polarity. Accumulated evidence indicates that adverse RhoC signaling affects angiogenesis and EMT processes, and is linked with cancer metastasis in different malignant tumors [89]. In 2017, Wu et al. reported that ABHD11-AS1 drives EOC progression through the activation of RhoC-mediated signaling pathway [30]. Moreover, the overexpression of ABHD11-AS1 lncRNA in OC cell lines upregulated RhoC levels, along with its downstream targets P70s6k, MMP2, and BCL-xL. In contrast, the silencing of ABHD11-AS1 through targeted siRNAs had opposite effects, and similar phenotypes to that of RhoC knockdown. Additionally, the RNA pull-down assay results showed that ABHD11-AS1 directly interacts with RhoC, and the knockdown of RhoC inhibited the oncogenic effect of ABHD11-AS1 [30]. Thus, the RhoC signaling pathway is a potential target of ABHD11-AS1 to drive tumor-promoting outcomes in OC.

#### 3.2.4. Regulation of the Cell Cycle

LncRNAs are widely expressed in human cells, and regulate various biological processes such as proliferation, apoptosis, differentiation, and the cell cycle. A high-content RNAi screen identified several lncRNAs involved in genomic stability and cell division [90]. The mammalian cell cycle is tightly regulated by cyclin-dependent kinases (CDKs), related retinoblastoma protein (RB), and p53 signaling pathways. Moreover, the cell cycle checkpoints play a critical role in maintaining cellular integrity [91]. Recent evidence indicates that lncRNAs are involved in modulating essential cell cycle regulators such as cyclins, CDKs, pRB, p53, and CDK inhibitors directly, or transcription factors that control CDKs/cyclins expression [92]. ABHD11-AS1 is one such lncRNA that regulates the cell cycle and contributes to cancer development and progression. Depletion of ABHD11-AS1 levels resulted in the downregulation of cell proliferation genes CDK2 and cyclin B1 in GC cell lines (MGC-803 and BGC-823) [18]. Overexpression of ABHD11-AS1 promoted cell cycle progression, whereas knockdown resulted in G0/G1 arrest through cyclin D1 in EC cell lines [35]. Accordingly, the suppression of ABHD11-AS1 lncRNA levels leads to G0/G1 phase arrest in CRC cell lines [28,29], PC cells via miR1231/cyclin E1 [26], and PTC cell lines [20,21]. These reports highlight that ABHD11-AS1 contributes to tumorigenesis by regulating cell cycle-related pathways.

### 3.3. Epigenetic Alterations

Epigenetics denotes the heritable changes in gene expression without modifications in DNA sequence. The epigenetic mechanisms include DNA methylation, histone modifications, chromatin remodeling, and non-coding RNAs. For several years, epigenetic dysregulation has contributed significantly to the development and progression of different human diseases, notably cancer. The epigenetic regulators are exploited as effective therapeutic targets for the treatment of cancer, and many molecules are in clinical development [93]. The potential epigenetic mechanisms that are critical for the transcriptional dysregulation of ABHD11-AS1 in human cancers are discussed below.

#### 3.3.1. Dysregulation of Histone Modifications by ABHD11-AS1 in Cancers

Among different epigenetic alterations, histone modification, such as methylation, has been known to be critical for various biological processes in all eukaryotes. Histone methylation on a few residues, such as H3K4, H3K36, and H3K79, induces gene transcription, while methylation on H3K9, H3K27, and H4K20 inhibits gene expression [94,95]. The lncRNAs affect target gene transcription by modulating histone methylation via histone methyltransferases or demethylases, and recruitment of chromatin-modifying complexes such as polycomb repressive complex 2 (PRC2) to the chromatin region [96,97]. One such lncRNA is ABHD11-AS1, which was reported to bind with EZH2 (Enhancer of Zeste Homolog 2), a catalytic subunit of PRC2 complex which maintains transcriptional repression through methylation of ‘Lys-9’ (H3K9me) and ‘Lys-27’ (H3K27me) of histone H3 in cancer [23,31]. In NSCLC, ABHD11-AS1 negatively regulated the expression of *KLF4* (Kruppel-Like Factor 4) transcription factor through the recruitment of EZH2, and thus H3K27me3 at its promoter to promote the Warburg effect [23]. Similarly, ABHD11-AS1 contributed to the progression of OC through repression of *TIMP2* expression via EZH2-mediated H3K27me3 at its promoter [31,32]. ABHD11-AS1 knockdown induced the expression of target genes *KLF4* and *TIMP2* with a significant reduction in the occupancy of EZH2 and H3K27me3 at their promoters [23,31]. The current evidence indicates that dysregulation of histone modifications is one of the potential mechanisms through which ABHD11-AS1 lncRNA promotes the development and progression of different cancers.

#### 3.3.2. Enhanced Stability of ABHD11-AS1 Transcript by N6-methyladenosine (m^6^A) Modification

Post-transcriptional modification of RNA gained the attention of researchers due to its emerging role both in physiological and pathological processes. N6-methyladenosine (m^6^A) modification is the most common form of dynamic and reversible RNA methylation in mammals. The m^6^A methylation accounting for 0.1–0.4% of adenosines in total RNA is modified [98]. The high-throughput profiling revealed that m^6^A sites are enriched in 3′-UTR and near stop codons [99]. The aberrant m^6^A methylation levels result in disease conditions, and significantly affect tumorigenesis and tumor progression. The dynamic equilibration in m^6^A levels were achieved by coordinated interaction of different enzymes, such as m^6^A methyltransferases (writers), m^6^A demethylases (erasers), and proteins that recognize m^6^A RNA modification (readers). The methyltransferase-like 3 (METTL3) is an important component of the classical complex of writers [100]. Very recently, Xue et al. have profiled m^6^A levels by methylated RNA immunoprecipitation sequencing (MeRIP-Seq), and discovered that ABHD11-AS1 lncRNA transcript gained m^6^A modification in NSCLC cells (H1299), in comparison to normal cells [23]. The bioinformatic analysis indicated METTL3 motif in m^6^A methylation on ABHD11-AS1 sequences. Moreover, overexpression of METTL3 significantly increased the m^6^A modification levels, and enhanced the stability of the ABHD11-AS1 lncRNA transcript. Therefore, it was concluded that METTL3-mediated m^6^A modification as a potential mechanism through which the ABHD11-AS1 is stably overexpressed, and contributes to the progression of NSCLC [23].

## 4. Conclusions and Perspectives

**Figure 5:**
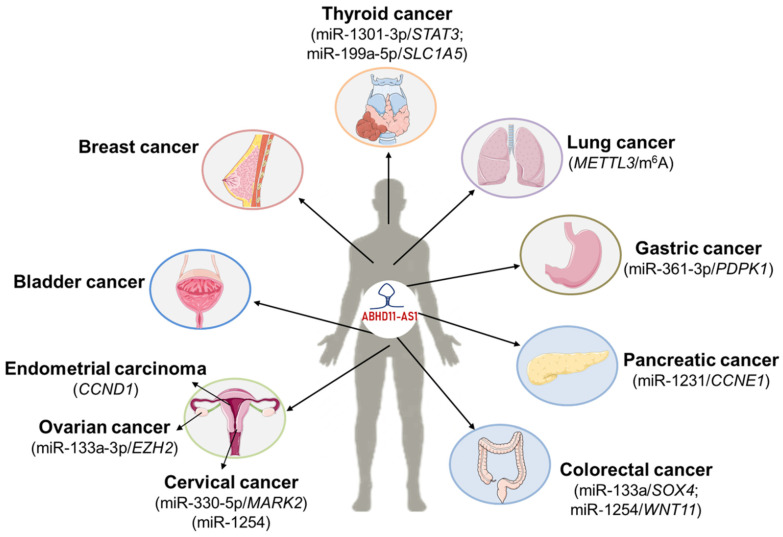
The role of ABHD11-AS1 lncRNA in the development and prognosis of different malignant tumors. The potential targets of ABHD11-AS1 in the progression of cancers were indicated. The artworks were adopted from https://smart.servier.com/ (Accessed on 14 October 2021).

LncRNAs are aberrantly expressed in human malignancies and exploited as biomarkers for tumor diagnosis, metastasis, invasion, and treatment. One such lncRNA with oncogenic potential is ABHD11-AS1, which has gained a lot of attention in recent years due to its significant role in human cancers. This review summarizes the studies that reported the molecular mechanisms through which ABHD11-AS1 exerts its tumor-promoting effects in malignant tumors, and serves as a biomarker for the early diagnosis and treatment of cancers (Figure 5). ABHD11-AS1 was upregulated and correlated positively with disease stage, metastasis, and inversely linked to overall survival rate in different cancers. ABHD11-AS1 mediates cancer-promoting effects by modulating different signaling pathways such as PI3K/Akt, EGFR, and RhoC in malignant tumors. In addition to the signaling pathways, epigenetic modifications such as histone methylation play an essential role in ABHD11-AS1 driven tumor development, progression, and metastasis. Similar to most lncRNAs, ABHD11-AS1 sponges multiple miRNAs by acting as ceRNA to modulate the transcription of oncogenic target genes. Recently, it was found that METTL3-mediated m^6^A modification on ABHD11-AS1 enhances the stability of its transcript, and thus contributes to the development of lung cancer. In a few studies, ABHD11-AS1 was established as circulatory lncRNA, and its levels in body fluids serve as an essential biomarker for the early diagnosis of PC and PTC. The ABHD11-AS1 levels in gastric juice could serve as a biomarker for the early diagnosis of gastric cancer. Altogether, ABHD11-AS1 has excellent potential to serve as a molecular marker for early diagnosis, treatment, and improving human cancers’ overall prognosis. Considering the available literature, the authors warrant further mechanistic studies employing advanced technologies such as CRISPR/Cas9, RIP, ChIP, ChIRP, and transcriptomics to delineate the functions and molecular mechanisms of ABHD11-AS1 lncRNA in human malignancies. The development of gene-targeted therapies could exploit ABHD11-AS1 as a potential therapeutic target for cancer treatment.

## Figures and Tables

**Table 1:** Summary of studies describing oncogenic role of ABHD11-AS1 lncRNA in different cancers.

Tumor Type	Sample Type	Patient/Control Size	Expression	Clinical Correlation	Function	Biomarker	Relevant to Prognosis	Overall Survival	Year	Reference
GC	Tissue	75/75	Upregulated	Degree of differentiation, Lauren type		Diagnosis			2014	[10]
	Tissue	73/37	Upregulated						2016	[16]
	Gastric juice	39/45	Upregulated	Gender, tumor size, tumor stage, Lauren type, blood CEA levels		Diagnosis/ Early diagnosis				
	Plasma	10/10	No change						2018	[17]
	Tissue	41/41	Upregulated		Proliferation, apoptosis				2020	[18]
PTC	Tissue	82/82	Upregulated	TNM stage, lymph node metastasis, tumor infiltration	Proliferation, apoptosis, migration		Negative correlation	Poor	2018	[19]
	Tissue	80/80	Upregulated		Proliferation, colony formation, migration, invasion, apoptosis	Diagnosis			2019	[20]
	Serum	64/50	Upregulated	Tumor diameter, lymph node metastasis	Proliferation, apoptosis	Diagnosis	Negative correlation	Poor	2021	[21]
	Tissue	98/98	Upregulated	Lymph node metastasis	Proliferation, invasion, migration	Diagnosis			2022	[22]
NSCLC	Tissue	40/40	Upregulated	TNM stage	Proliferation, Warburg effect		Negative correlation	Poor	2020	[23]
PC	Tissue	147/147	Upregulated	TNM stage, distant metastasis, and tumor differentiation	Proliferation, colony formation, migration, invasion, apoptosis	Prognosis	Negative correlation	Poor	2018	[24]
	Plasma	114/46	Upregulated			Diagnosis/ Early diagnosis	Negative correlation	Poor	2019	[25]
	Tissue/ TCGA	179/171	Upregulated		Proliferation, migration, invasion, apoptosis			Poor	2020	[26]
CRC	Tissue	132/132	Upregulated	TNM stage, lymph node metastasis	Proliferation, colony formation, migration, invasion, apoptosis		Negative correlation	Poor	2018	[27]
	Tissue	53/53	Upregulated		Proliferation, colony formation, invasion				2019	[28]
	Tissue	60/60	Upregulated		Proliferation, migration, invasion, apoptosis				2021	[29]
OC	Tissue	51/13	Upregulated	Tumor stage, Degree of differentiation	Proliferation, apoptosis, invasion, migration				2017	[30]
	Tissue	53/53	Upregulated		Proliferation, invasion, migration, colony formation				2019	[31]
	Tissue	50/50	Upregulated	Tumor stage, lymph node metastasis	Proliferation, apoptosis, invasion, migration			Poor	2021	[32]
Bladder Cancer	Tissue	66/66	Upregulated	TNM stage, Histological grade, tumor invasion depth	Proliferation, apoptosis, migration,				2017	[33]
BC	Tissue	79/79	Upregulated	No association				Same	2021	[34]
EC	Tissue	89/27	Upregulated		Proliferation, apoptosis, invasion, migration				2018	[35]
CC	Cell lines		Upregulated		Proliferation, apoptosis, colony formation, invasion, migration				2021	[36]
	Tissue/ Serum	72/78	Upregulated		Proliferation, apoptosis, invasion, migration	Diagnosis/ Prognosis	Negative correlation	Poor	2022	[37]

**Table 2:** List of miRNAs targeted by ABHD11-AS1 lncRNA in human cancers.

S.No.	miRNA Name	Target Gene	Cancer Type	Reference
1	miR-361-3p	3-Phosphoinositide Dependent Protein Kinase 1 (*PDPK1*)	GC	[18]
2	miR-1301-3p	Signal Transducer and Activator of Transcription 3 (*STAT3*)	PTC	[19]
3	miR-199a-5p	Solute Carrier Family 1 Member 5 (*SLC1A5*)		[20]
4	miR-330-5p	Microtubule Affinity Regulating Kinase 2 (*MARK2*)	CC	[36]
5	miR-1254	-		[37]
6		Wnt Family Member 11 (*WNT11*)	CRC	[28]
7	miR-133a	SRY-Box Transcription Factor 4 (*SOX4*)		[27]
8	miR-1231	cyclin E1 (*CCNE1*)	PC	[26]
9	miR-133a-3p	-	OC	[32]
